# MiR-548c impairs migration and invasion of endometrial and ovarian cancer cells via downregulation of Twist

**DOI:** 10.1186/s13046-016-0288-0

**Published:** 2016-01-13

**Authors:** Xiaochun Sun, Manhua Cui, Aichen Zhang, Lingling Tong, Kun Wang, Kai Li, Xue Wang, Ziqian Sun, Hongye Zhang

**Affiliations:** Department of Obstetrics and Gynecology, China-Japan Union Hospital of Jilin University, Changchun, 130021 China; Department of Obstetrics and Gynecology, the Second Hospital of Jilin University, Changchun, 130041 China

**Keywords:** microRNA, miR-548c, EMT, Endometrial cancer, Ovarian cancer

## Abstract

**Background:**

MicroRNAs (miRNAs) are a class of small non-coding RNAs, which post-transcriptionally repress the expression of genes involved in cancer initiation and progression. Although some miRNAs that target many signaling pathways (also called universe miRNAs) are supposed to play a global role in diverse human tumors, their regulatory functions in gynecological cancers remain largely unknown. We investigated the biological role and underlying mechanism of miR-548c (one universe miRNA) in endometrial and ovarian cancer.

**Methods:**

The effects of miR-548c overexpression on cell proliferation, migration and invasion were studied in endometrial and ovarian cancer cells. TWIST1 (Twist) was identified as a direct miR-548c target by western blot analysis and luciferase activity assay. The expression of miR-548c and Twist were examined by qRT-PCR in endometrial and ovarian cancer tissues.

**Results:**

Here, we report that miR-548c is down-regulated in endometrial and ovarian cancer tissues when compared to normal tissues, and our meta-analysis reveal that decreased miR-548c expression correlates with poor prognosis in endometrial cancer patients. We show that in endometrial and ovarian cancer cells, ectopic expression of miR-548c significantly inhibits whereas knockdown of miR-548c dramatically induces cancer cell proliferation, migration and invasion. By using luciferase reporter assay, we demonstrate that *Twist*, a known oncogene in endometrial and ovarian cancers, is a direct target of miR-548c. Furthermore, the expression of Twist partially abrogates the tumor suppressive effects of miR-548c on cell migration and invasion.

**Conclusion:**

These findings suggest that miR-548c directly downregulates Twist, and provide a novel mechanism for Twist upregulation in both endometrial and ovarian cancers. The use of miR-548c may hold therapeutic potential for the treatment of Twist-overexpressing tumors.

## Background

Metastasis is the main cause of death among patients with endometrial cancer (EC, the most frequently diagnosed gynecologic malignancy) cbl^1^, ^2^] and ovarian cancer (OC, the most lethal gynecological cancer) [3]. The initial steps of metastasis include the detachment and migration of cancer cells and subsequent invasion into surrounding tissues [4]. Epithelial-to-mesenchymal transition (EMT) is considered a key step in metastasis of endometrial and ovarian cancer [1, 5, 6], which enhances the migratory and invasive capacity of tumor cells to facilitate malignant progression. Therefore, understanding the molecular mechanisms that mediate migration, invasion and EMT process of EC and OC cells could identify novel molecular targets for future treatments of these cancers.

*Twist1* (Twist) is a critical oncogene that is overexpressed and plays important roles in EMT induction in EC and OC [1, 7, 8]. High Twist expression positively correlated with deep myometrial invasion and poor outcome in EC [9]. Aberrant expression of Twist in OC correlated with advanced tumor stage and predicted a poor clinical outcome [10, 11]. MicroRNAs (miRNAs) are a class of small non-coding RNA molecules that regulate gene expression via interactions with the 3’-untranslated regions (UTRs) of mRNAs targets, causing translational suppression or mRNA decay. MiRNAs control metastatic progression [12, 13], and act as either tumor suppressors or oncogenes in EC and OC [1, 14]. Although a number of miRNAs, such as miR-106b and miR-543, have been reported to suppress the expression of Twist and EC cell invasion [15, 16], miRNAs affecting Twist expression in EC and OC is still not completely understood. Recently, two distinct classes of microRNAs: universe miRNAs (for example, miR-34a) that regulate many signaling pathways, as well as intra-pathway miRNAs that target multiple genes within a single signaling pathway, have been identified [17]. Universe microRNAs are predicted to affect more targets/signaling pathways, and might play a global role in tumor cells [17]. Of interest, several universe miRNAs (including miR-548a/b/c, etc.) belong to the same miR-548 family [17] and have been implicated in tumorigenesis [18, 19], however their regulatory functions in gynecological cancers remain largely unknown.

In this study, we investigated the biological functions and underlying mechanism of miR-548c in EC and OC, and provide in vitro evidence that miR-548c inhibits migration and invasion in both EC and OC cells by directly targeting Twist. We further demonstrate that miR-548c expression inversely correlates with Twist expression in EC and OC tissues and the downregulation of miR-548c is associated with poor prognosis in EC patients.

## Methods

### Cell culture and transient transfection

The human EC cell lines (RL95-2 and HEC-1) and human OC cell lines (SKOV-3 and OVCAR3) were obtained from Chinese Academy of Sciences Committee on Type Culture Collection Cell Bank, Shanghai, China. These cells were cultured in DMEM/F12 medium (Invitrogen, Shanghai) or PRMI-1640 medium (Gibco, Carlsbad, CA, USA) supplemented with 10 % fetal bovine serum (FBS, Invitrogen, Shanghai). MiR-548c mimic (40 nM), antisense miR-548c inhibitor (40 nM), Twist siRNA (5 nM) and respective negative controls were purchased from Ambion (TX, USA), and were transfected using Lipofectamine 2000 (Invitrogen, CA, USA), according to the manufacturer’s instructions. Transient transfection of Twist cDNA plasmids (OriGene, MD, USA) were performed with Lipofectamine Plus reagent (Invitrogen, CA, USA) according to the manufacturer’s protocol.

### RNA extraction and real-time qRT-PCR

Total RNA was extracted using TRIzol reagents (Invitrogen, CA, USA). The miRNA qRT**-**PCR was performed using the NCode miRNA qRT**-**PCR analysis (Invitrogen, CA, USA). Forward primers for miRNA detection are the exact sequence of mature miR-548c-3p. For mRNA analysis, RT reaction was carried out using 100 ng total RNA with the PrimeScript RT reagent kit (Takara, Japan). Real-time qRT-PCR was performed using the Takara SYBR Premix Ex Taq II (Takara, Japan). Primers used for the amplification of *E-cadherin* [20], *N-cadherin* [20], *CD133* [21], *MMP-9* [22] and GAPDH [22] have been previously described. All miRNA and mRNA quantification data were normalized to GAPDH. Results were given as the fold change relative to controls.

### Cell proliferation assay and apoptosis assay

To evaluate cell proliferation, 5 × 10^3^ cells were plated in 96-well plates for 24 h, and then transfected with miRNA mimic/inhibitor with or without Twist siRNA or Twist cDNA vector. At 72 h, cell proliferation was determined by the Cell Counting Kit-8 (Dojindo, Japan). The absorbance was determined at 450 nm using a microplate reader, and data were expressed as the percentage of absorbance relative to controls. For cell apoptosis assay, cells were transfected for 72 h and apoptotic cells were identified using the DeadEnd Colorimetric TUNEL System kit (Promega, WI, USA), following the manufacturer’s instructions. Cell apoptosis was quantified as the numbers of apoptotic cells found in 15 random fields. The ratio of dead/total cell number was calculated.

### Cell migration and invasion assay

Cells were transfected for 24 h and then seeded into upper chamber of Boyden chambers coated with or without Matrigel as described previously [23–25]. After incubation for 24 h, migration and invasion were stained and counted under a light microscope. Relative migration and invasion activities were expressed as the fold change over respective controls.

### Western blot

Cells were harvested 48 h after transfections. Whole-cell protein extracts were prepared using the M-Per Mammalian Protein Extraction Reagent (Pierce Biotechnology, MA, USA) according to the manufacturer’s instructions. Total proteins (40 μg) were loaded onto 10 % SDS-PAGE for immunoblots with antibodies to Twist (Abcam, ab50887) and GAPDH (Santa Cruz, sc-47724). Primary antibodies were used at a dilution of 1:1000.

### Luciferase activity assay

The *Twist* 3’-UTR luciferase vectors was obtained from OriGene (Rockville, MD, USA). A quick-change site-directed mutagenesis kit (Stratagene, CA, USA) was used to mutate the miR-548c-binding site. Luciferase activity was measured at 24 h after transfection using the dual-luciferase reporter assay system (Promega, WI, USA) as previously reported [15].

### Benign and tumor tissues

50 pairs of primary EC and adjacent non-tumor endometrial tissues, 60 epithelial OC tissues and 20 normal epithelial ovarian tissues were collected in the China-Japan Union Hospital of Jilin University, China. This study was approved by the Ethics Committee of China-Japan Union Hospital of Jilin University and conducted according to the principles of the Declaration of Helsinki. The patients signed a written inform consent for the procedures in the study. Samples were snap-frozen immediately at –80 °C, and total RNA was isolated using TRIzol reagents.

### Statistical analysis

Results are presented as mean ± s.e.m. from at least three independent experiments performed in triplicate. 2-tailed Student’s *t*-test was used for statistical analysis. *P*-values < 0.05 were defined as significant.

## Results

### MiR-548c is downregulated in endometrial and ovarian cancer tissues and decreased miR-548c levels associates with worse prognosis in patients with endometrial cancer

**Fig. 1:**
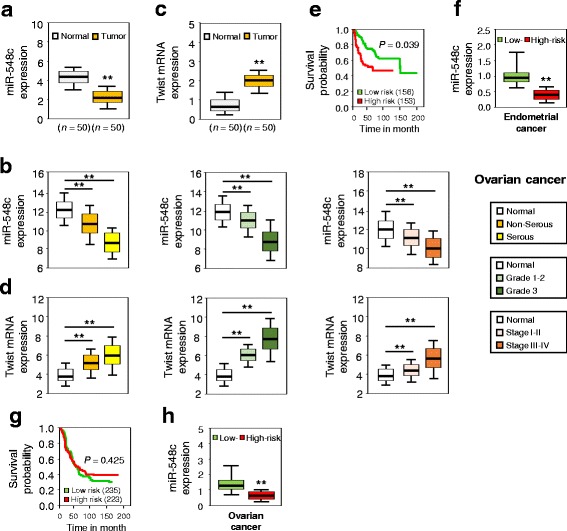
MiR-548c is downregulated in endometrial and ovarian cancer tissues and decreased miR-548c levels associates with worse prognosis in patients with endometrial cancer. Box plots showing decreased expression of miR-548c (**a**, **b**) and increased *Twist* (**c**, **d**) mRNA expression in 50 ECs and matched adjacent normal tissues (top), and in 60 OCs compared to 20 normal ovarian tissues (bottom), assessed by the quantitative real-time RT-PCR (qPCR) assays. Of note, downregulation of miR-548c (**b**) and upregulation of *Twist* (**d**) was detected in serous, advanced stage and grade 3 OC samples. Kaplan-Meier survival curves of EC (**e**) and OC (**g**) patients in The Cancer Genome Atlas (TCGA) data set were created using the SurvMicro database. In ECs, high-risk patients exhibited significantly lower levels of miR-548c compared to the low-risk patients (**f**). **h** MiR-548c expression between high-risk and low-risk OCs (TCGA). ^**^ *P* < 0.01

To examine the levels of miR-548c in EC tissues, we measured the endogenous miR-548c expression by real-time quantitative RT-PCR (qPCR) in 50 EC samples and their adjacent normal tissues. Intriguingly, miR-548c was significantly decreased in ECs compared with adjacent normal tissues (Fig. 1a). We also analyzed miR-548c expression by qPCR in clinical samples of 60 OC tissues (Table 1) compared to 20 normal ovarian tissues. Decreased miR-548c expression was detected in OC tissues relative to normal ovarian tissues, and the expressions of miR-548c were significantly lower in serous, advanced stage and grade 3 tumor samples compared with non-serous, early stage and grade 1/2 tumor samples (Fig. 1b), indicating that the deregulation of miR-548c may play a critical role in EC or OC development.

**Table 1:** Clinical characteristics of 60 ovarian cancer patients

Features	Cases (%)
Age (years)	
≤55	32 (53.3 %)
>55	28 (46.7 %)
Clinical stage	
I–II	19 (31.7 %)
III–IV	41 (68.3 %)
Pathological grade	
1–2	24 (40.0 %)
3	36 (60.0 %)
Histotype	
Serous	40 (66.7 %)
Non-serous	20 (33.3 %)

To investigate whether the downregulation of miR-548c is related with prognosis of EC or OC patients, we used the SurvMicro web tool [26] to investigate the relationship between miR-548c levels and prognosis of cancer patients. The analysis of published data on EC and OC from The Cancer Genome Atlas (TCGA) revealed that the low expression levels of miR-548c were associated with very poor overall survival in EC patients compared with those patients whose ECs express a high level of miR-548c (Fig. 1e and f). Although our meta-analysis of OCs using TCGA data did not show a clear separation between high-risk OCs and low-risk OCs based on miR-548c expression (*P* = 0.425), OC patients whose tumors display low miR-548c expression tend to have shorter survival (Fig. 1g and h). Thus, these data uncover an association between the loss of miR-548c expression and poor survival of EC patients, and indicate that miR-548c repression might be important for EC/OC growth and progression.

### MiR-548c inhibits proliferation, migration and invasion of EC and OC cells

**Fig. 2:**
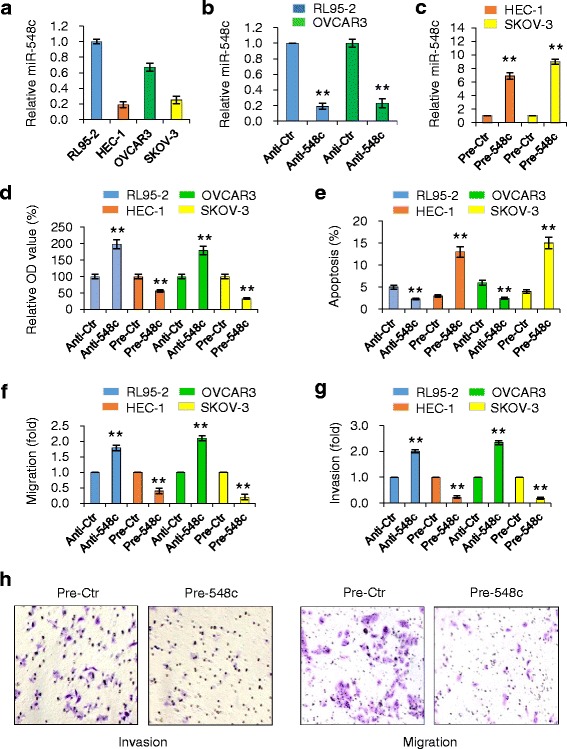
MiR-548c inhibits proliferation, migration and invasion of EC and OC cells. **a** Relative expression of miR-548c in two EC cell lines (RL95-2 and HEC-1) and OC cell lines (OVCAR3 and SKOV-3 was determined by qPCRs. Data is presented as the fold-change in expression compared with RL95-2. **b** qPCR analysis of miR-548c expression in RL95-2 and OVCAR3 cells transfected with anti-miR-548c inhibitor (anti-548c) or control inhibitor (anti-ctr). **c** miR-548c levels were measured by qPCRs in HEC-1 or SKOV-3 cells after overexpression of pre-miR-548c mimic (pre-548c) or control mimic (pre-ctr). Cell proliferation assay (**d**), cell apoptosis assay (**e**), transwell migration (**f**) and matrigel invasion (**g**) assays were performed in indicated cells after knockdown or overexpression of miR-548c, respectively. **h** Representative images from cell migration and invasion assays performed in SKOV-3 cells. ^**^ *P* < 0.01

To select appropriate model for functional studies, we first evaluated miR-548c expression in two EC cell lines (RL95-2 and HEC-1) and OC cell line SKOV-3 using qPCRs. We found that invasive HEC-1 and SKOV-3 cells express much lower levels of miR-548c, compared with less invasive RL95-2 and OVCAR3 cells (Fig. 2a). The downregulation of miR-548c in cancer tissues and invasive cancer cells implicated that it might have the tumor suppressive roles in the tumorigenesis and metastasis of EC and OC. To test this, we transiently transfected anti-miRNA inhibitor against miR-548c (anti-548c) or control inhibitor (anti-ctr) into RL95-2 or OVCAR3 cells, and then validated the depletion of miR-548c expression by qPCR assays (Fig. 2b). Furthermore, the ectopic overexpression of miR-548c in HEC-1 and SKOV-3 cells was achieved by transient transfection of pre-miR-548c mimic (pre-548c) (Fig. 2c). Then, cell proliferation was measured by the Cell Counting Kit-8 assay. The downregulation of miR-548c in RL95-2 and OVCAR3 cells resulted in greater proliferation rate, whereas forced miR-548c expression significantly reduced proliferation of HEC-1 and SKOV-3 cells compared with the control mimic (pre-ctr) (Fig. 2d). To determine the effect of miR-548c on cell apoptosis, we examined apoptosis-associated DNA fragmentation by using a colorimetric TUNEL staining assay. MiR-548c knockdown decreased RL95-2 or OVCAR3 cell death, however miR-548c overexpression caused apoptotic cell death of HEC-1 and SKOV-3 cells (Fig. 2e). These results suggest that miR-548c inhibits EC and OC cell proliferation and triggers cell apoptosis.

To verify whether miR-548c affect the progression and metastasis of EC and OC, we evaluated the effect of either knockdown or overexpression of miR-548c on the migratory and invasive abilities of these cells. Transwell migration and invasion assays demonstrated that downregulation of miR-548c expression significantly promoted migration and invasion of RL95-2 and OVCAR3 cells (Fig. 2f and g). In contrast, transient overexpression of miR-548c in HEC-1 and SKOV-3 cells significantly impaired in vitro cell migration and invasion (Fig. 2f, g and h). Together, these data support that miR-548c acts as a tumor suppressors to inhibit tumor growth, migration and invasion in both EC and OC cells.

### Twist is a direct target of MiR-548c in EC and OC cells

**Fig. 3:**
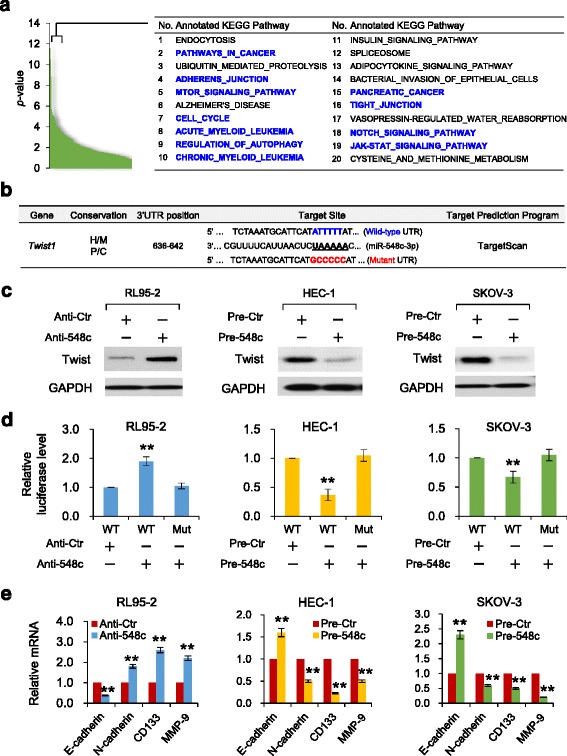
Twist is a direct target of miR-548c in EC and OC cells. **a** *In silico* prediction and molecular pathway enrichment analysis indicate that miR-548c-regulated genes are preferentially involved in pathway associated with tumor growth and metastasis. The top-20 ranking KEGG pathways were listed. **b** The putative conserved miR-548c-binding site in the *Twist* 3’-UTR. Mutation was generated in the binding site. **c** Western blotting analysis of Twist expression in RL95-2, HEC-1 or SKOV-3 cells after knockdown or overexpression of miR-548c. **d** Indicated cells were transfected with reporter constructs containing either wild-type (WT) *Twist*, or *Twist* 3’-UTR with mutation (Mut), along with miR-548c mimic, anti-miR-548c inhibitor or negative controls, respectively. Relative luciferase activity was assayed. **e** qPCR analysis of EMT, invasion and stemness-related genes in RL95-2 upon miR-548c knockdown, or in HEC-1 or SKOV-3 cells after overexpression of miR-548c. ^**^ *P* < 0.01

To explore gene targets and molecular pathways that could be mediated by miR-548c, we performed computational target prediction using TargetScan and conducted *in silico* enrichment analysis of miR-548c-target genes in KEGG pathways using miRSystem [27]. Of note, miR-548c-regulated genes can potentially modulate numerous well-known pathways associated with tumor growth and metastasis, including pathways in cancer, mTOR pathway, adherens junction, cell cycle, acute myeloid leukemia, pancreatic cancer, notch signaling pathway and jak-stat signaling pathway (Fig. 3a), indicating the possibility that miR-548c has broad influence over diverse oncogenic pathways. These findings are consistent with our results (Fig. 2), supporting the roles of miR-548c to suppress multiple malignant phenotypes of EC and OC cells including proliferation, migration and invasion.

Because *Twist* was identified as a potential target of miR-548c (Fig. 3b), we speculated that *Twist* serves as a direct target of miR-548c. Our western blot analysis suggested that the introduction of anti-548c led to an increase in Twist protein level in RL95-2 cells, while pre-548c resulted in the opposite effects in HEC-1 and SKOV-3 cells (Fig. 3c). To test whether miR-548c repression is mediated through the predicted binding site, the reporter plasmid containing putative miR-548c responsive site was transfected with anti-548c or anti-ctr into RL95-2 cells. The downregulation of miR-548c enhanced *Twist* 3’-UTR luciferase reporter activity compared with the negative control (Fig. 3d, left), suggesting that miR-548c suppresses translation of Twist. To verify that miR-548c directly represses *Twist* on its 3’-UTR, we cotransfected pre-548c or pre-ctr with the *Twist* 3’-UTR reporter into HEC-1 and SKOV-3 cells. The ectopic expression of pre-548c caused a repression on luciferase activity as compared with pre-ctr (Fig. 3d, middle and right). To further evaluate the interaction between miR-548c and *Twist* 3’-UTR, we generated *Twist* 3’-UTR mutated for miR-548c binding site and performed the luciferase assays upon either miR-548c overexpression or knockdown. Mutant 3’-UTR reporter activity was resistant to the addition of anti-548c or pre-548c (Fig. 3d), confirming that *Twist* is a direct target of miR-548c, and binding of miR-548c to the 3’-UTR of *Twist* mRNA is sufficient to reduce Twist protein levels.

Twist binds the *E-cadherin* promoter and represses E-cadherin transcription, whereas directly activating N-cadherin expression [28, 29]. In addition, the EMT induction has been linked to the emergence of a cancer stem cell (CSC)-like phenotype [30]. Consistent with this notion, sorted CD133^+^ EC cells have been shown to demonstrate greater tumor initiating capacity than CD133^−^ cells [31], and CSC-like cells obtained from SKOV-3 cells express higher levels of CD133 [32]. Matrix metalloproteinase 9 (MMP-9) is a downstream target of Twist in tumor cells [33]. We test whether miR-548c regulates these genes in cancer cells used in this study. By using qPCR, we found a significant decrease in the expression of *E-cadherin* and an increased expression in *N-cadherin*, *CD133* and *MMP-9* levels in anti-548c-transfected RL95-2 cells (Fig. 3e). In contrast, miR-548c overexpression in HEC-1 and SKOV-3 cells significantly induced *E-cadherin* levels, but inhibited the expression of *N-cadherin*, *CD133* and *MMP-9* (Fig. 3e). Furthermore, we used qPCR to examine the mRNA expression of *Twist* in EC and OC tissues. As expected, *Twist* levels was markedly elevated in EC samples when compared to adjacent normal tissues (Fig. 1c). We also observed significantly increased *Twist* expression in OCs relative to normal ovarian tissues, and found higher levels of *Twist* in serous, advanced, high-grade ovarian cancers (Fig. 1d). Altogether, these data are consistent with our hypothesis that increased Twist expression in EC and OC is a functional consequence of miR-548c repression (at least in part).

### Twist mediates miR-548c-reduced cancer cell migration and invasion

**Fig. 4:**
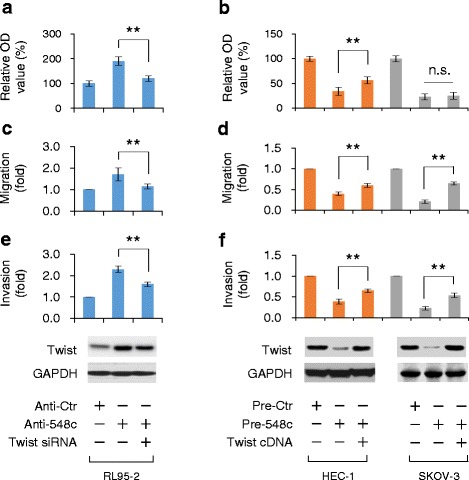
Twist mediates miR-548c-reduced cancer cell proliferation, migration and invasion. RL95-2, HEC-1 and SKOV-3 cells were transfected with anti-548c, pre-548c, Twist siRNA or Twist cDNA vector lacking 3’-UTR as indicated, and were assayed for cell proliferation (**a**, **b**), migration (**c**, **d**) and invasion (**e**, **f**) (top). Western blot analysis of Twist protein levels (bottom). ^**^ *P* < 0.01

To further study whether miR-548c can attenuate oncogenic phenotypes in EC and OC cells through downregulation of Twist, we performed cell proliferation, migration and invasion assays using EC or OC cells transduced with anti-548c or pre-548c, along with either Twist siRNA or Twist cDNA expression plasmid without 3’-UTR sequence, which renders the escape from miR-548c regulation. Anti-548c-induced RL95-2 cell proliferation, migration and invasion were significantly abrogated by Twist siRNA (Fig. 4a, c and e). On the other hand, overexpression of Twist cDNA largely rescued pre-548c-suppressed proliferation, migration and invasion in HEC-1 cells (Fig. 4b, d and f, left). Although expression of Twist cDNA restored SKOV-3 cell migration and invasion that were reduced by pre-548c (Fig. 4d and f, right), cell proliferative phenotype was not significantly restored by Twist overexpression in SKOV-3 cells (Fig. 4b, right), consistent with a previous report showing that Twist confers migratory and invasive potential, but does not affect proliferation of OC cells [8]. Collectively, these data support the mechanistic roles for miR-548c in impairing migration and invasion of EC and OC cells by targeting Twist.

## Discussion

Targeting of metastasis-related miRNAs in human tumors may offer potential therapeutic strategies for these diseases [34, 35]. Recent findings have identified miR-548c family members as universe miRNA, and illustrated the possibility that these miRNAs exert broad effects on many features of tumor cells through a broad range of targets and pathways [17]. Although one such miRNA, miR-548c, has been shown to inhibit cancer cell growth [19], its cellular functions, underlying mechanisms and clinical significance have not been comprehensively studied in gynecological cancers. Here, we found that miR-548c was under-expressed in both EC and OC samples, compared to their non-tumor counterparts. At the molecular level, miR-548c represses migration and invasion of EC and OC cells partially by targeting Twist. We demonstrate that miR-548c expression was inversely correlated with *Twist* level in EC and OC tissues, and reduced miR-548c expression was associated with poor prognosis in EC patients. These findings reveal for the first time that in EC and OC cells, miR-548c is a tumor-suppressive miRNA that inhibits multiple malignant features of the metastatic cascade.

*Twist* has been known as a novel oncogene overexpressed in diverse tumors [36], and activation of Twist inhibits apoptosis and promotes the induction of EMT, cancer stemness, proliferation, angiogenesis and vasculogenic mimicry in human tumor cells, all of which contributes to metastasis [37, 38]. Several upstream regulators and downstream effector of Twist have been reported [37], however our knowledge of the detailed molecular mechanisms involved in Twist overexpression, especially the epigenetic regulation of Twist expression, is still incomplete. Here, we reported a previously unidentified mechanism whereby loss of miR-548c expression is partially responsible for increased Twist levels in EC and OC, two of the most common gynecologic cancers.

Twist has been shown to upregulate the expression of some miRNAs, such as miR-10b [39] and miR-424 [40]. Twist promotes the expression of miR-10b by directly binding to the promoter of miR-10b, which initiates tumor invasion and metastasis in breast cancer [39]. Furthermore, miR-424, another miRNA that can be induced by Twist, drives EMT-like phenotypes in breast cancers [40]. The impact of Twist on miR-548c expression in EC and OC cells warrants further investigation.

MiR-548c was reported to be an independent prognostic factor for breast cancer. Patients with a good prognosis presented higher intratumoral expression of miR-548c [41]. Consistently, our meta-analysis suggested that high expression of miR-548c in EC and OC appears to correlate with better survival in patients with EC and OC, implying that miR-548c may be a new promising prognostic factor in these cancers. Since miR-548c directly regulates Twist in EC and OC cells, this miRNA might also represent an attractive target for the treatment of Twist-overexpressing tumors.

## Conclusions

Our findings provide a novel mechanism by which the repression of miR-548c increases Twist levels to stimulate cancer cell migration and invasion of both EC and OC cells. Our results indicate that miR-548c is a potential therapeutic target in EC and OC.
